# A deep learning methodology for improved breast cancer diagnosis using multiparametric MRI

**DOI:** 10.1038/s41598-020-67441-4

**Published:** 2020-06-29

**Authors:** Qiyuan Hu, Heather M. Whitney, Maryellen L. Giger

**Affiliations:** 1 Committee on Medical Physics, Department of Radiology, The University of Chicago, 5841 S Maryland Ave., Chicago, IL MC202660637 USA; 2 Department of Physics, Wheaton College, Wheaton, IL USA

**Keywords:** Breast cancer, Cancer imaging, Computational science, Computer science, Information technology, Software, Statistics

## Abstract

Multiparametric magnetic resonance imaging (mpMRI) has been shown to improve radiologists’ performance in the clinical diagnosis of breast cancer. This machine learning study develops a deep transfer learning computer-aided diagnosis (CADx) methodology to diagnose breast cancer using mpMRI. The retrospective study included clinical MR images of 927 unique lesions from 616 women. Each MR study included a dynamic contrast-enhanced (DCE)-MRI sequence and a T2-weighted (T2w) MRI sequence. A pretrained convolutional neural network (CNN) was used to extract features from the DCE and T2w sequences, and support vector machine classifiers were trained on the CNN features to distinguish between benign and malignant lesions. Three methods that integrate the sequences at different levels (image fusion, feature fusion, and classifier fusion) were investigated. Classification performance was evaluated using the receiver operating characteristic (ROC) curve and compared using the DeLong test. The single-sequence classifiers yielded areas under the ROC curves (AUCs) [95% confidence intervals] of AUC_DCE_ = 0.85 [0.82, 0.88] and AUC_T2w_ = 0.78 [0.75, 0.81]. The multiparametric schemes yielded AUC_ImageFusion_ = 0.85 [0.82, 0.88], AUC_FeatureFusion_ = 0.87 [0.84, 0.89], and AUC_ClassifierFusion_ = 0.86 [0.83, 0.88]. The feature fusion method statistically significantly outperformed using DCE alone (*P* < 0.001). In conclusion, the proposed deep transfer learning CADx method for mpMRI may improve diagnostic performance by reducing the false positive rate and improving the positive predictive value in breast imaging interpretation.

## Introduction

Breast magnetic resonance imaging (MRI) has been reported to be a highly sensitive imaging modality for breast cancer detection and characterization^1^. Dynamic contrast-enhanced (DCE)-MRI offers morphological and functional lesion information with excellent sensitivity and variable specificity for breast cancer diagnosis^2^. Moderate specificity may lead to unnecessary subsequent patient work-up and biopsies, which may contribute to anxiety in patients awaiting biopsy results that indicate benignity. To overcome this limitation and assess more functional data, approaches to examining other MRI sequences alongside DCE-MRI images have been implemented in the routine clinical interpretation of breast MRI exams over recent decades^2, 3^. This approach is defined as multiparametric MRI (mpMRI), in which T2-weighted (T2w) MRI is a commonly used additional sequence. Studies have shown that the incorporation of T2w sequence during interpretation is useful in the differential diagnosis of benign and malignant lesions^4–6^. For example, fibroadenomas, a type of benign lesions that can exhibit similar contrast agent enhancement to that of malignant lesions on T1-weighted DCE-MRI, usually have high signal intensity on T2w images compared with malignant lesions^4^.

In order to assist radiologists in the interpretation of diagnostic imaging, computer-aided diagnosis (CADx) systems continue to be developed with artificial intelligence (AI) techniques to potentially improve the accuracy of evaluating suspicious breast lesions^7^. Multiparametric CADx schemes using multiple MRI protocols have also started to be explored as MRI technology advances^8–11^. In this study, we propose and evaluate the performance of three AI-integrated multiparametric CADx methods that incorporate the complementary information provided in DCE and T2w MRI protocols in the task of distinguishing between benign and malignant breast lesions. In addition, we compare them with the performances of the two single-sequence-based methods, i.e., DCE and T2w.

We employ a deep transfer learning methodology that extracts and pools low- to mid-level features using a pretrained convolutional neural network (CNN) and perform classification using a support vector machine (SVM). We explore integrating the information from the DCE and T2w MRI sequences at three different levels of the classification framework, namely via (i) input of the multiparametric images directly to the CNN (image level), (ii) input of the CNN features extracted from DCE and T2w into a multiparametric classifier (feature level), and (iii) aggregation of the outputs of the DCE SVM and T2w SVM (classifier output level). We believe that this is the first comprehensive study of mpMRI schemes, and our methodologies demonstrate strong potential in utilizing information from mpMRI to estimate the probability of breast lesion malignancy without the need for preprocessing, image registration, large datasets, or long training times.

## Methods

### Study participants

The study was approved by the Institutional Review Board (IRB) of the University of Chicago and followed Health Insurance Portability and Accountability Act (HIPAA)-compliant protocols. The database was retrospectively collected under the above-mentioned protocols, and all procedures were conducted in accordance with relevant guidelines and regulations. The requirement for informed consent was waived because all clinical information and images in this study were de-identified to the investigators. The MRI exams in the database were consecutively acquired over the span of eight years, from 2005 to 2013, imaged at a single institution. Exclusion criteria included MRI studies that did not exhibit a visible lesion, lesions that did not have validation of the final diagnosis, or lesions that could not be clearly allocated to either the benign or malignant category. A total of 927 unique breast lesions from 616 women (mean age 55.0 ± 12.8 years; age range 23–89 years) were ultimately included in this study.

**Figure 1:**
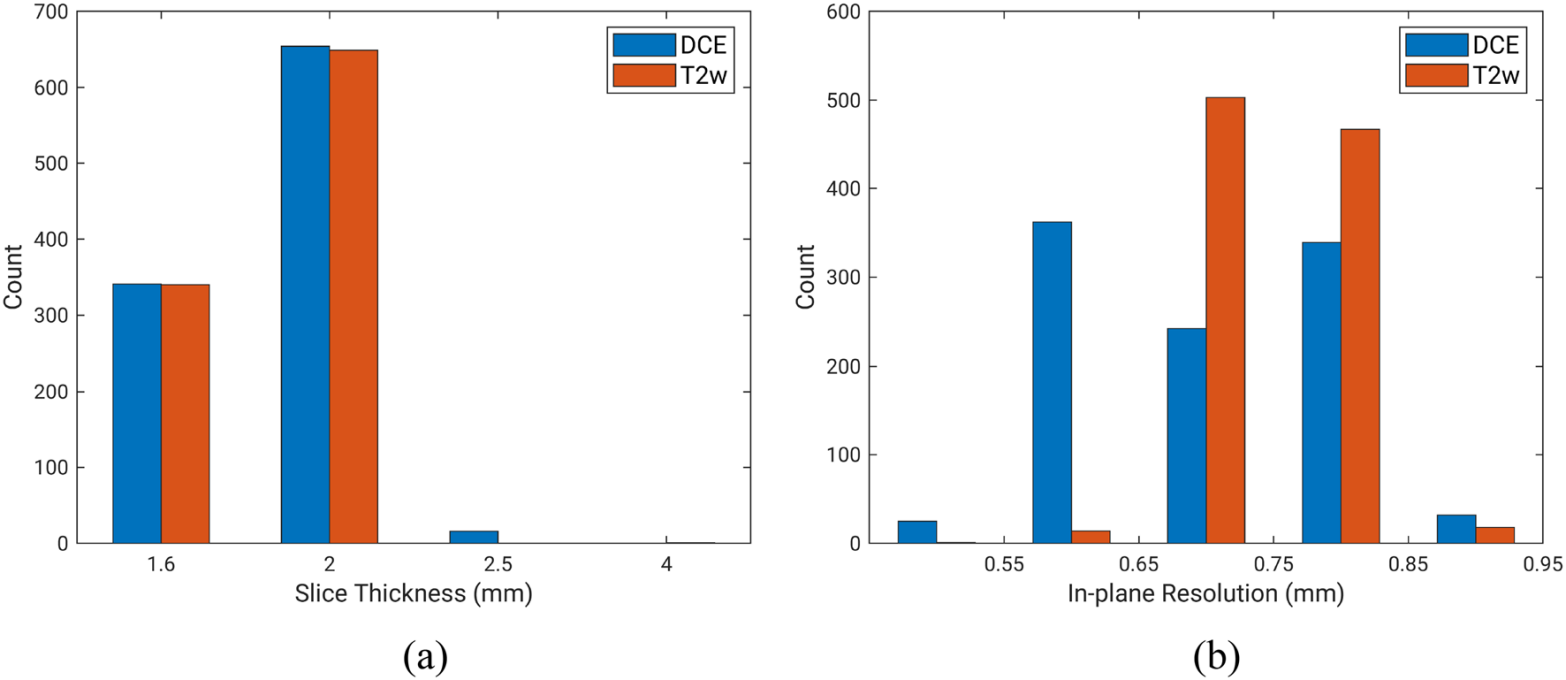
Distribution of slice thickness and in-plane resolution of the dynamic contrast-enhance (DCE) sequences and T2-weighted (T2w) sequences in the multiparametric MRI database.

Of all lesions, 199 were benign (21%) and 728 were malignant (79%). For all lesions clinically categorized at MRI as Breast Imaging Reporting and Data System (BI-RADS) category 4, 5, or 6, malignant/benign status was confirmed by histopathology. For all lesions clinically categorized at MRI as BI-RADS category 2 or 3, benign diagnosis was confirmed by MRI follow-up of at least 24 months. Lesions were thus labeled as either benign or malignant based on pathology and radiology reports. Images in the database were using either 1.5 T or 3 T Philips Achieva scanners with a T1-weighted spoiled gradient sequence and a T2-weighted turbo spin echo sequence without fat suppression. Therefore, each MR study contained a DCE-MRI sequence and a T2w MRI sequence acquired during the same exam. The temporal resolution for each dynamic acquisition in the DCE sequence was 60 s. Image slice thickness varied across the dataset and across the two sequences. The slice thickness was consistent across the two sequences (i.e., DCE and T2w) in 96% of the exams, while the in-plane resolution was consistent across the two sequences in 46% of the exams. Figure 1 shows the distribution of slice thickness and in-plane resolution of images in this dataset. Clinical characteristics of the dataset are detailed in Table 1.

**Table 1:** Clinical characteristics of the dataset.

Benign/malignant prevalence	Benign: 199 (21.5)
	Malignant: 728 (78.5)
Age (years): mean ± standard deviation	55.0 ± 12.8
	Unknown: 97
**Benign lesion characteristics**	
Lesion size (mm)	Mean: 8.86
	Median: 7.33
	Range: 3.38–42.8
Lesion subtypes	Fibroadenoma: 60 (30.2)
	Columnar change: 15 (7.5)
	Papilloma: 13 (6.5)
	Parenchyma tissue: 12 (6.0)
	Fibrotic tissue: 10 (5.0)
	Hyperplasia: 8 (4.0)
	Cystic change: 6 (3.0)
	Fat necrosis: 5 (2.5)
	Other: 27 (13.6)
	Unknown: 43 (21.6)
**Malignant lesion characteristics**	
Lesion size (mm)	Mean: 17.9
	Median: 14.9
	Range: 3.37–73.7
Lesion subtypes	IDC: 147 (20.2)
	DCIS: 120 (16.5)
	IDC + DCIS: 359 (49.3)
	ILC: 31 (4.3)
	ILC mixed: 26 (3.6)
	Other: 33 (4.5)
	Unknown: 12 (1.6)
Estrogen receptor status	Positive: 410 (56.3)
	Negative: 128 (17.6)
	Unknown: 190 (26.1)
Progesterone receptor status	Positive: 352 (48.4)
	Negative: 184 (25.3)
	Unknown: 192 (26.4)
HER-2 status	Positive: 87 (12.0)
	Negative: 404 (55.5)
	Equivocal: 5 (0.7)
	Unknown: 232 (31.9)

Numbers in parentheses are percentages. Patient age is summarized on a patient basis, and lesion information (malignancy status and subtypes) is summarized on a lesion basis.

For some subjects, only the decade of age was available (e.g., “60 s”) as part of the patient information deidentification process. In these situations, the middle of the decade was used for the calculation of the mean subject age. Lesion size is measured by the effective diameter, i.e., the greatest dimension of a sphere with the same volume as the lesion.

*IDC* invasive ductal carcinoma, *DCIS* Ductal carcinoma in situ, *ILC* invasive lobular carcinoma, *HER-2* human epidermal growth factor receptor 2.

### Single-sequence methods

**Figure 2:**
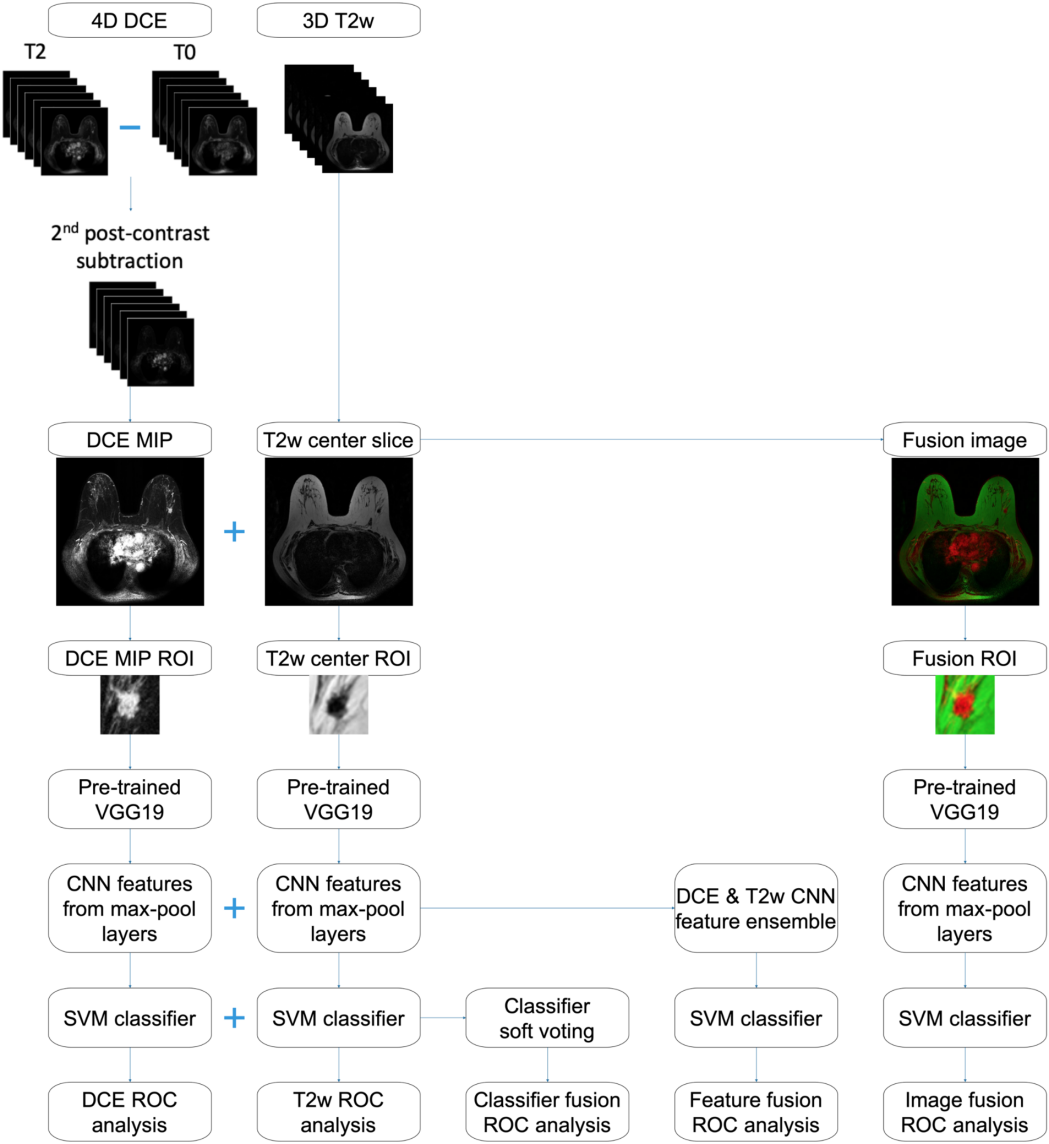
Lesion classification pipeline based on diagnostic images. Information from dynamic contrast-enhanced (DCE) and T2-weighted (T2w) MRI sequences are incorporated in three different ways: *image fusion*—fusing DCE and T2w images to create RGB composite image, *feature fusion*—merging convolutional neural network features extracted from DCE and T2w as the support vector machine (SVM) classifier input, and *classifier fusion*—aggregating the probability of malignancy output from the DCE and T2w classifiers via soft voting. *MIP* maximum intensity projection, *ROI* region of interest, *ROC* receiver operating characteristic.

Figure 2 schematically shows the machine learning classification and evaluation process for both single-sequence and mpMRI schemes.

Lesions were segmented using a fuzzy C-means method requiring only the manual indication of a seed-point^12^. Lesion segmentations were not directly used as input to the CNN, but enabled automatic region of interest (ROI) construction described below. To capture the 4D (volumetric and temporal) characteristics of the lesions from the DCE sequence, maximum intensity projection (MIP) images of the second postcontrast subtraction DCE-MRI series were used as the input to a deep learning network^13^. The second post-contrast timepoint was chosen because the BI-RADS atlas defines the initial phase of enhancement as the first two minutes after contrast administration, which has diagnostic utility for distinguishing benign and malignant breast lesions^14^. From the T2w sequence of each lesion, the slice that contained the largest lesion area according to the automatic lesion segmentation was selected as the representative center slice, which was used as the input to a deep learning network. The T2w center slice was rescaled using bicubic interpolation to match the in-plane resolution of its corresponding DCE sequence. To avoid confounding contributions from distant voxels, a ROI around each lesion was cropped from the image to use in the subsequent classification process. The ROI size was chosen based on the maximum dimension of each lesion and was held constant across sequences. A small part of the parenchyma, three pixels wide around the lesion, was included in each ROI. Appropriate shifts in the coordinates were applied to ensure that the DCE and T2w ROIs were cropped from the same location relative to the lesion.

Through transfer learning, CNN features were extracted separately from the ROIs of the DCE subtraction MIPs and the ROIs of the T2w center slices using the publicly available VGG19 model^15^, pretrained on ImageNet^16^. The pretrained VGG19 network, which takes three-channel (red, green, and blue, or RGB) input images, has previously been shown to be useful in transfer learning for breast lesion analyses^13,17,18^. For the single-sequence DCE and T2w image datasets, the ROIs were grayscale and were duplicated across the three channels. Feature vectors were extracted at various network depths from the five max-pooling layers of the VGGNet. These features were then average-pooled along the spatial dimensions and normalized with Euclidian distance. The pooled features were then concatenated to form a CNN feature vector of 1,472 features for a given lesion^17,18^.

Nonlinear SVM classifiers with Gaussian radial basis function kernel were trained on the CNN features to differentiate between benign and malignant lesions (Python Version 3.4.2, Python Software Foundation)^19^. SVM was chosen over other classification methods due to its ability to handle sparse high-dimensional data, which is an attribute of the CNN features. To address the problem of class imbalance (i.e., due to the 79% cancer prevalence), a misclassification penalty for cases in the malignant (or benign) class was assigned to be inversely proportional to the malignant (or benign) class prevalence in the training data.

### Evaluation of single-sequence methods

Each SVM classifier was trained and evaluated using nested fivefold cross-validation, where the inner cross-validation was used for hyperparameter tuning and the outer cross-validation was used for training and testing, resulting in a 64%/16%/20% split into independent training, validation, and test sets, respectively. Class prevalence was held constant across the five cross-validation folds, and all lesions from the same patient were kept together in the same fold in order to eliminate the impact of using correlated lesions for both training and testing. The training set was standardized to zero mean and unit variance, and the test set was standardized using those statistics of the corresponding training set. Principal component analysis fit on the training set was applied to both training and test sets to reduce feature dimensionality^20^.

Within each training/validation fold in the outer cross-validation loop, two SVM hyperparameters, namely the scaling parameter *γ* and the regularization parameter *C*, were optimized on a grid search with an internal fivefold cross-validation^21^. Prediction scores were transformed to posterior probabilities of malignancy (PMs) assuming a scaled prevalence of 50%^22^. The predictions on the five test folds were aggregated for classification performance evaluation.

Classifier performances were evaluated using receiver operating characteristic (ROC) curve analysis, with area under the ROC curve (AUC) serving as the figure of merit^23,24^. Standard errors and 95% confidence intervals (CIs) of the AUCs were calculated by bootstrapping the posterior PMs (2000 bootstrap samples)^25^. Other clinical metrics, including sensitivity, specificity, positive predictive value (PPV), and negative predictive value (NPV), for each classifier were also reported. These metrics were calculated at the optimal operating point on the ROC curve determined by minimizing *m = (1 − sensitivity)*^*2*^ *+ (1 − specificity)*^*2*^^11^.

### Multiparametric methods

We explored integrating information from both the DCE and T2w MRI sequences at three different levels of the classification framework, as illustrated in Fig. 2. The three mpMRI schemes are referred to as image fusion, feature fusion, and classifier fusion.

**Figure 3:**
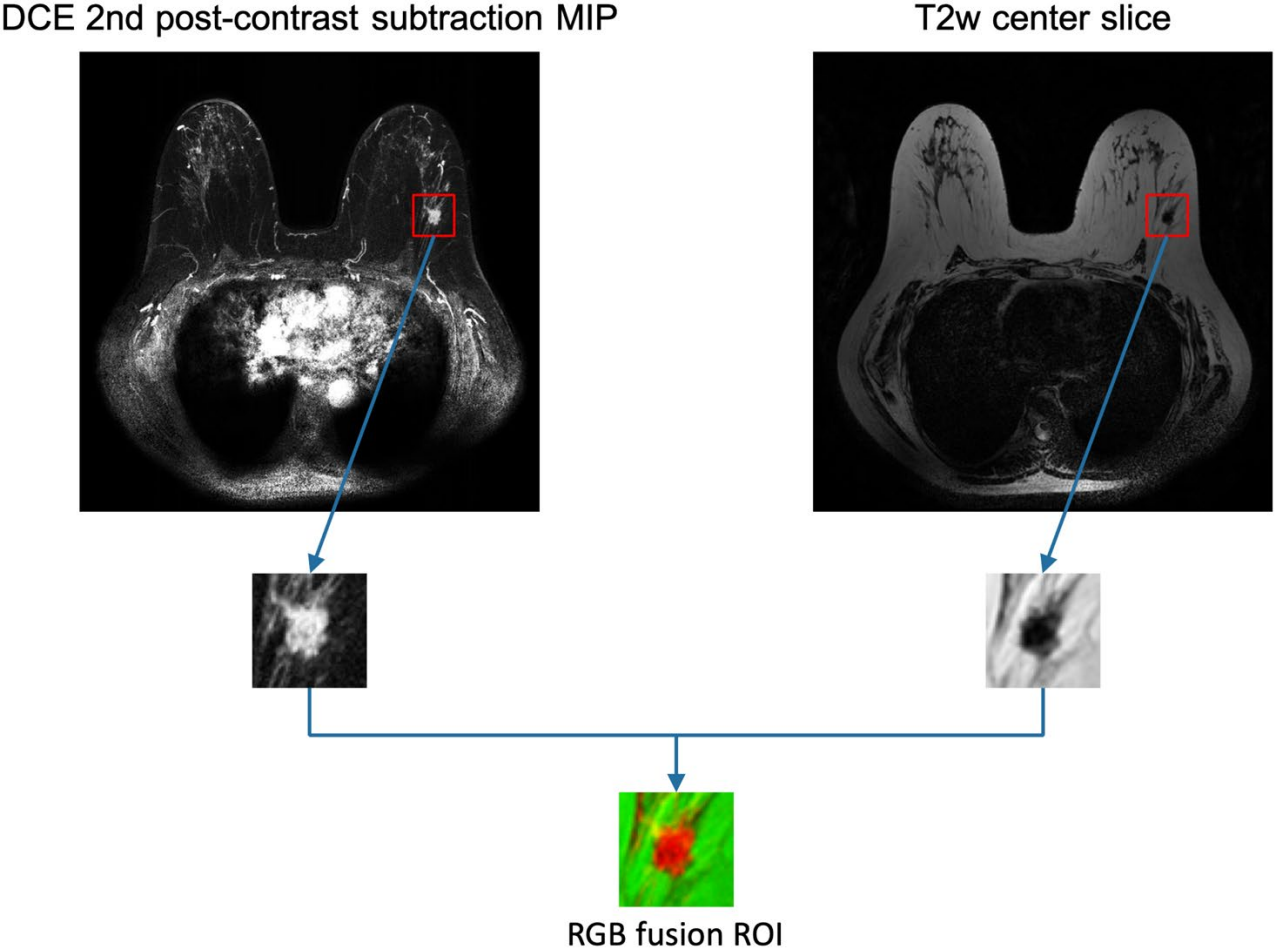
Example input images. A dynamic contrast-enhanced (DCE)-MRI transverse second post-contrast subtraction maximum intensity projection (MIP) and a T2-weighted (T2w)-MRI transverse center slice are shown with their corresponding regions of interest (ROIs) extracted. The RGB fusion ROI is created by inputting the DCE ROI into the red channel and the T2w ROI into the green channel.

For the input *image fusion* scheme, a three-channel RGB fusion image was constructed for each lesion by inputting the DCE MIP into the red channel, the T2w center slice into the green channel, and leaving the blue channel of the VGGNet blank. A composite ROI was cropped from the fusion image, which was then input into the pretrained VGG19 network for feature extraction. Figure 3 includes an example to illustrate the process of ROI extraction from MRI images and creating RGB fusion ROIs. The classifier training process then followed the single-sequence methods to predict PMs.

For the *feature fusion* scheme, CNN features extracted from DCE and T2w separately were included into an ensemble of features, which was then input to an SVM classifier. The classifier training process then followed the single-sequence methods to yield PMs.

For the *classifier fusion* scheme, PM outputs from the DCE-based and from the T2w-based single-sequence SVM classifiers were aggregated via soft voting. That is, the DCE and T2w PM outputs were averaged to yield prediction scores.

In the evaluation of each mpMRI scheme, the same evaluation method was used as for the single-sequence classifiers.

### Inter-sequence image registration

A preliminary study was performed to investigate whether image registration between DCE and T2w sequences would improve the performance of the proposed mpMRI classification schemes, especially the image fusion method. The T2w center slices were rescaled to match the in-plane resolution and then registered to the corresponding slice of the second post-contrast DCE image using a multi-modality rigid registration method that consists of translation and rotation^26, 27^. The same five classification mechanisms were evaluated after image registration.

### Statistical analysis

The AUCs from the three mpMRI classification schemes were statistically compared with those from the two single sequence classifiers using the DeLong test^28,29^. Bonferroni–Holm corrections were used to account for multiple comparisons^30^, and a corrected *P* < 0.05 was considered to indicate a statistically significant difference in performance. Equivalence testing was performed to assess if image registration had any effect on the classification performances^31^. An equivalence margin of difference in AUC = 0.05 was chosen *prima facie*.

Finally, to assess the performance reproducibility of the method, the highest performing classifier of the three mpMRI methods was trained and evaluated 100 times using different random seeds for the cross-validation split, and the mean and standard error of AUC was calculated from all the runs.

## Results

### Classification performance

**Figure 4:**
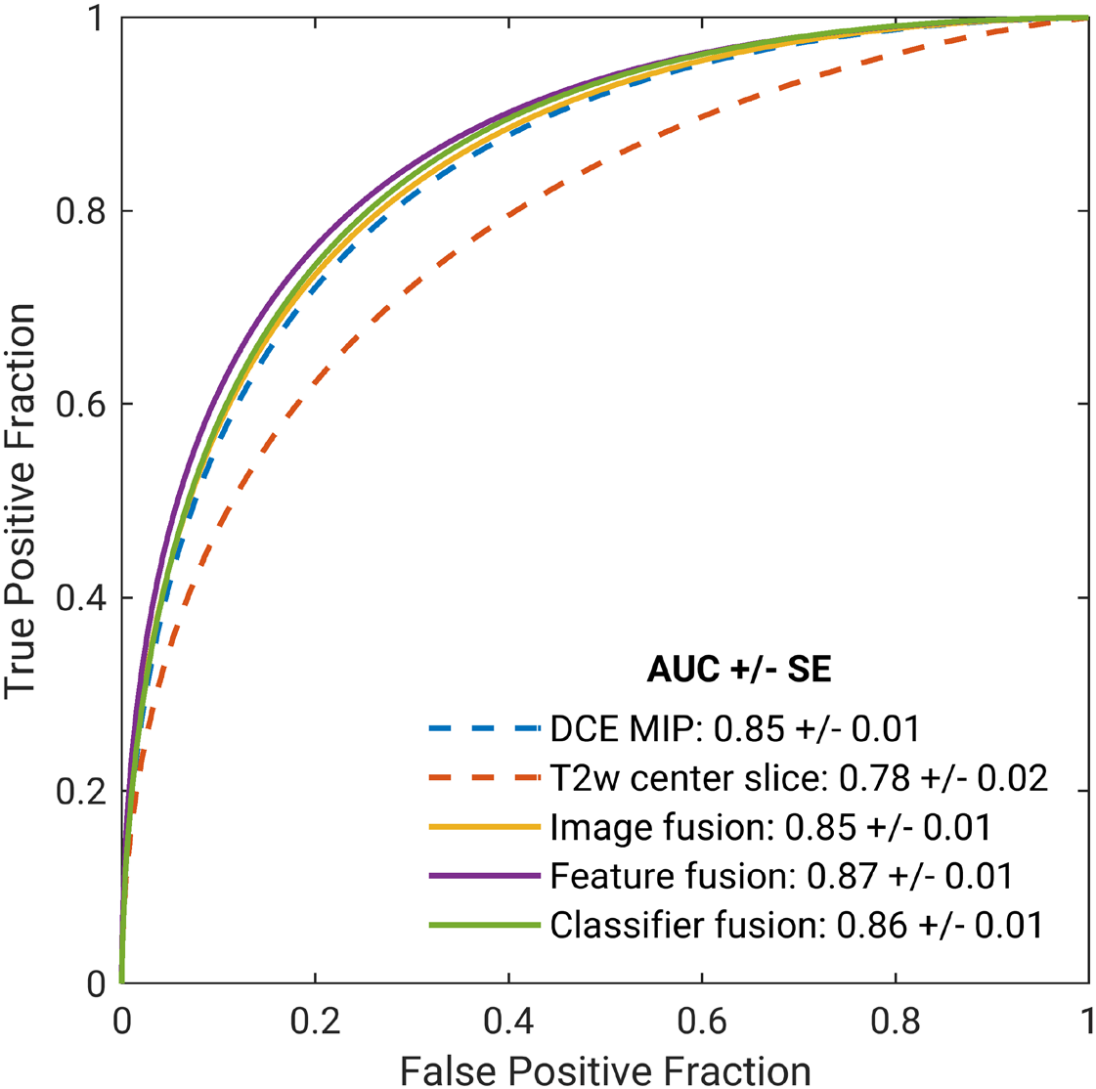
Fitted binomial receiver operating characteristic (ROC) curves for two single-sequence and three mpMRI classifiers using (i) convolutional neural network (CNN) features extracted from dynamic contrast-enhanced (DCE) subtraction maximum intensity projections (MIPs), (ii) CNN features extracted from T2-weighted (T2w) center slices, (iii) CNN features extracted from DCE and T2w fusion images, (iv) ensemble of features extracted from DCE and T2w images, and (v) probability of malignancy outputs from the DCE MIP and T2w classifiers aggregated via soft voting. The legend gives the area under the ROC curve (AUC) with standard error (SE) for each classifier scheme. T2w images were rescaled to match the in-plane resolution of their corresponding DCE sequences, but image registration was not performed.

Figure 4 presents the ROC curves for the five classification schemes without image registration, and Table 2 summarizes the classification performances as measured by AUC, sensitivity, specificity, PPV, and NPV. Note that the mpMRI classifiers achieved improvements in terms of all these metrics for classification performance. Table 3 shows the p-values and the 95% CIs for the comparisons of AUCs between the mpMRI and single-sequence classifiers. Among the three mpMRI classification schemes, while all of them yielded statistically significantly higher AUCs than using T2w alone, only the feature fusion method significantly outperformed using DCE alone in terms of AUC, and the other two methods, image fusion and classifier fusion, failed to demonstrate a statistically significant difference in AUCs compared with using DCE alone.

**Table 2:** Sensitivity, specificity, positive predictive value (PPV), negative predictive value (NPV), and area under the receiver operating characteristic curve (AUC) along with the 95% confidence interval (CI) for AUC for each classifier.

Classifier	DCE	T2w	Image fusion	Feature fusion	Classifier fusion
AUC [95% CI]	0.85 [0.82, 0.88]	0.78 [0.75, 0.81]	0.85 [0.82, 0.88]	0.87 [0.84, 0.89]	0.86 [0.83, 0.88]
Sensitivity (%)	75.9	69.8	76.5	77.9	77.6
Specificity (%)	76.5	72.7	77.1	78.5	77.1
PPV (%)	89.7	87.3	90.0	90.7	90.1
NPV (%)	54.2	47.3	55.0	56.9	56.2

Sensitivity and specificity presented are for the optimal operating point determined using a metric for cut-off value that minimizes *m = (1 − sensitivity)*^*2*^* + (1 − specificity)*^*2*^.

**Table 3:** Performance comparison for the five classifiers.

Classifier	DCE MIP	T2w center slice
Image fusion	*P* = 0.73 95% CI ∆AUC = [− 0.01, 0.02]	*P* < 0.001* 95% CI ∆AUC = [0.05, 0.09]
Feature fusion	*P* < 0.001* 95% CI ∆AUC = [0.01, 0.03]	*P* < 0.001* 95% CI ∆AUC = [0.06, 0.11]
Classifier fusion	*P* = 0.28 95% CI ∆AUC = [− 0.00, 0.02]	*P* < 0.001* 95% CI ∆AUC = [0.06, 0.09]

The classifier names are shown in the first row (single-parametric) and first column (multiparametric). P-value and 95% confidence interval (CI) of the difference in area under the receiver operating characteristic curves (AUCs) for each comparison are presented in the table, where each multiparametric classifier was compared with each single-parametric classifier using the DeLong test. P-values were corrected for multiple comparisons using Bonferroni–Holm corrections. Asterisks denote significance (*P* < 0.05) after accounting for multiple comparisons.

In assessing performance reproducibility, the mean and standard error of AUCs from 100 runs for the feature fusion classifier was 0.864 ± 0.003, indicating that the classification performance was very stable regardless of the random seed chosen.

**Figure 5:**
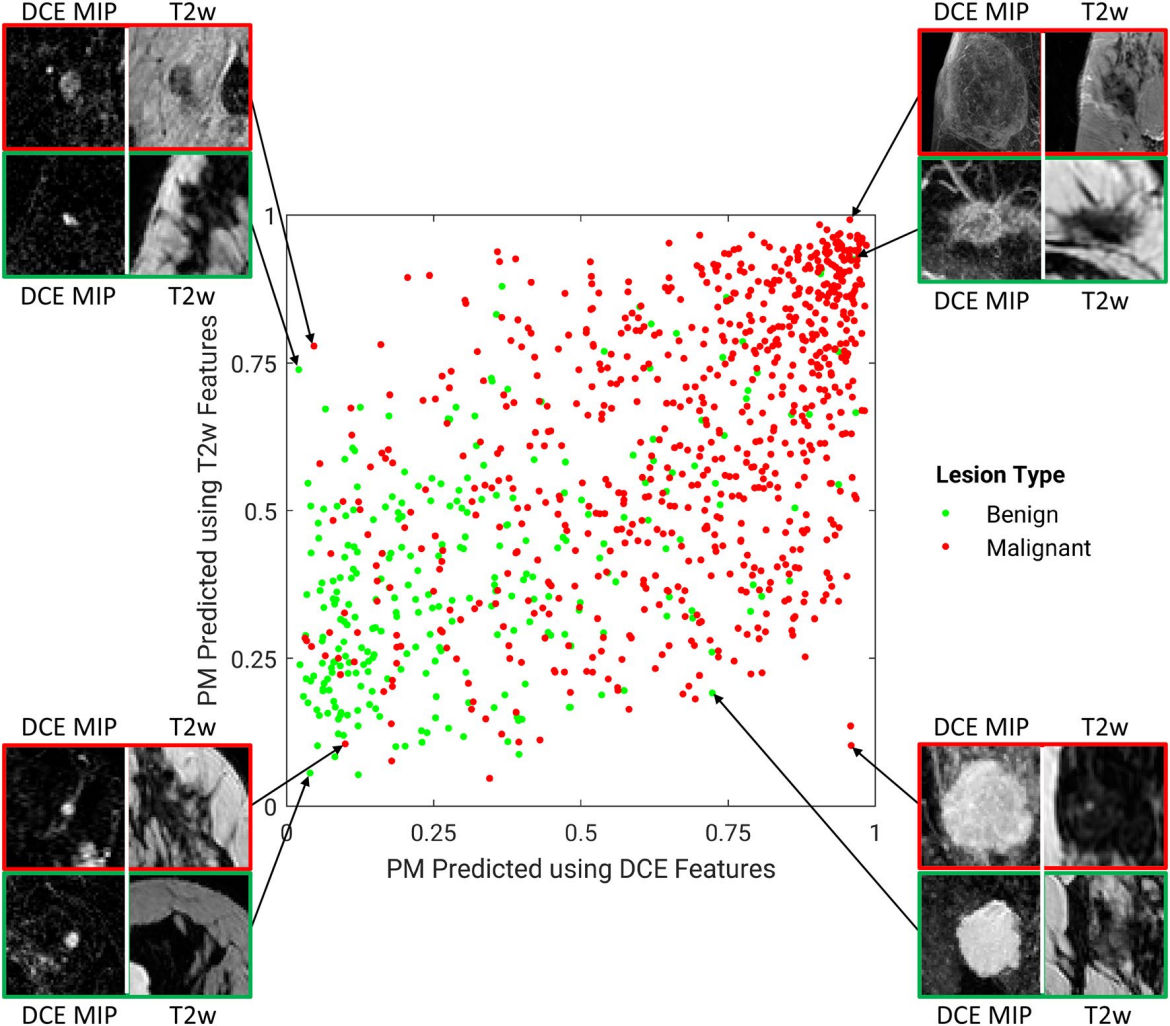
A diagonal classifier agreement plot between the T2-weighted (T2w) and dynamic contrast-enhanced (DCE) single-sequence classifiers. The x-axis and y-axis denote the probability of malignancy (PM) scores predicted by the DCE classifier and the T2w classifier, respectively. Each point represents a lesion for which predictions were made. Points along or near the diagonal from bottom left to top right indicate high classifier agreement; points far from the diagonal indicate low agreement. Examples of lesions on which the two classifiers were in extreme agreement/disagreement are also included.

**Figure 6:**
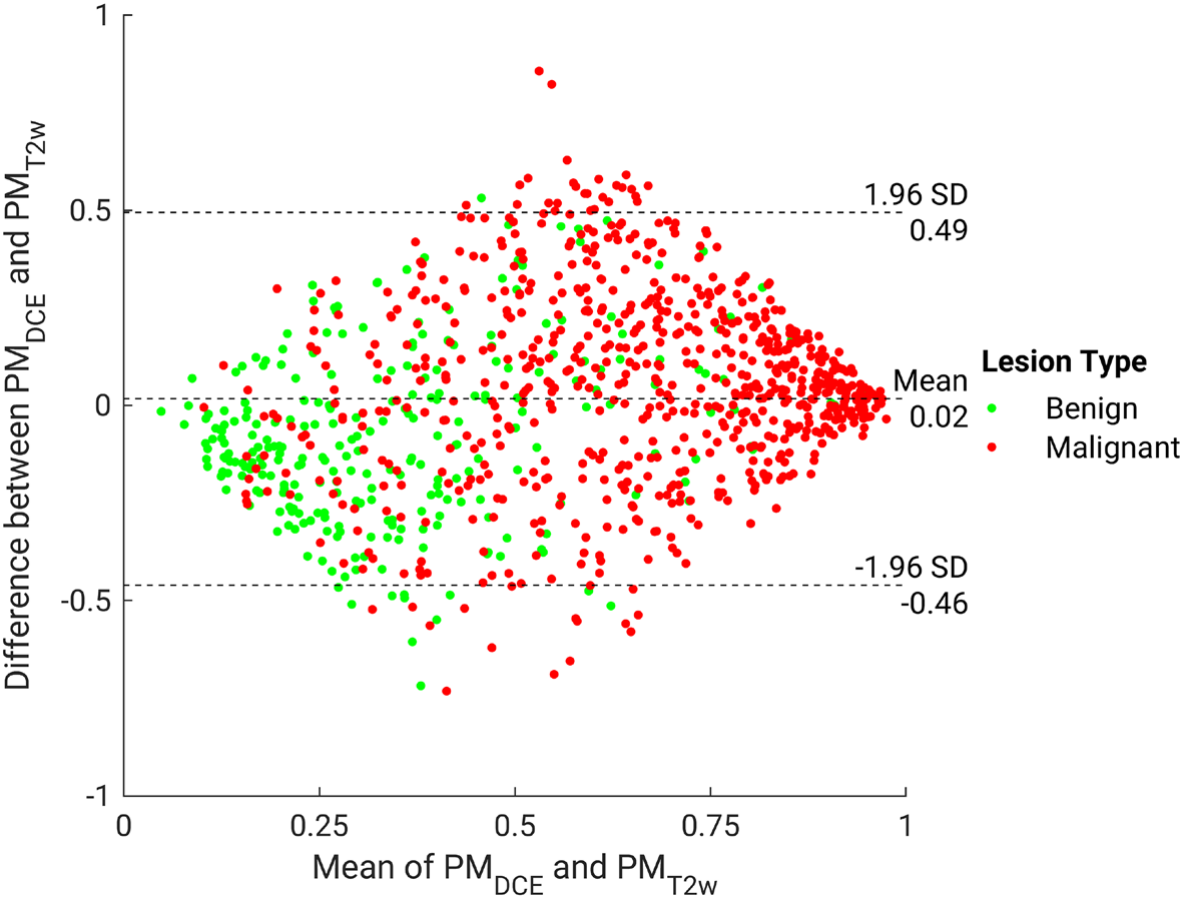
Bland–Altman plot illustrating classifier agreement between the dynamic contrast-enhanced (DCE) maximum intensity projection and T2-weighted (T2w)-based single-sequence classifiers. The y-axis shows the difference between the support vector machine output scores (predicted posterior probabilities of malignancy) of the two classifiers; the x-axis shows the mean of two classifiers’ outputs, which is also the probability of malignancy scores calculated in the classifier fusion method.

Figures 5 and 6 illustrate the comparison between the PMs predicted by the single-sequence classifiers using DCE and T2w. Figure 5 also shows example lesions on which these two classifiers agree or disagree. While the majority of benign and malignant lesions are separated from the other class, there appears to be moderate disagreement between the two classifiers, suggesting that a fusion technique could likely improve the predictive performance.

### Inter-sequence image registration

Performing inter-sequence rigid image registration did not have a significant effect on the classification performances of any classification scheme. Namely, the four classifiers affected by the registration (i.e., use information from T2w images) yielded AUC values of AUC_T2w_ = 0.79 ± 0.02 (95% CI [0.76, 0.82]), AUC_ImageFusion_ = 0.84 ± 0.01 (95% CI [0.81, 0.87]), AUC_FeatureFusion_ = 0.87 ± 0.01 (95% CI [0.84, 0.89]), and AUC_ClassifierFusion_ = 0.86 ± 0.01 (95% CI [0.83, 0.88]). Just as when T2w was not registered to DCE, while all three mpMRI classification schemes significantly outperformed using T2w alone, only feature fusion significantly outperformed using DCE alone. According to the 95% CIs of the difference in AUCs (∆AUCs) between performing inter-sequence image registration or not, image registration between T2w and DCE failed to show a statistically significant effect on the performance of any classifiers examined. In addition, equivalence testing demonstrated that whether image registration was performed or not yielded equivalent performance with an equivalence margin of ∆AUC = 0.05, chosen *prima facie*. Thus, all findings held regardless of whether image registration was employed or not, indicating that registration did not lead to a change in the performance of the mpMRI schemes.

## Discussion

The proposed convolutional neural network (CNN)-based multiparametric magnetic resonance imaging (mpMRI) computer-aided diagnosis (CADx) methods that take advantage of the complimentary information provided by dynamic contrast-enhanced (DCE) and T2-weighted (T2w) MRI protocols demonstrated potential to improve the performance of current CADx schemes in the task of distinguishing between benign and malignant breast lesions. Among the three mpMRI methods examined, the *feature fusion* method, i.e., using CNN features extracted from both DCE and T2w as the classifier input, significantly outperformed using DCE-MRI alone as in currently available CADx systems. The *image fusion* method, i.e., fusing the DCE and T2w images into one RGB image prior to input to the VGGNet, and the *classifier fusion* method, i.e., aggregating the probability of malignancy output from the DCE and T2w classifiers via soft voting, failed to show a statistically significant difference in performance compared with using DCE alone. All three mpMRI schemes statistically significantly outperformed using T2w alone. Furthermore, we demonstrated that image registration of the DCE and T2w images did not affect the classification performances when applied in addition to image resolution matching.

Training CNNs from scratch typically relies on massive datasets for training and is thus often intractable for medical research due to data scarcity. It has been shown that standard transfer learning techniques like fine-tuning or feature extraction based on ImageNet-trained CNNs can be used for CADx^32,33^. As a result, deep learning techniques have exhibited strong predictive performances on CADx tasks without requiring massive datasets^17,18,34–36^. Previous studies have investigated mpMRI CADx in distinguishing between malignant and benign lesions using human-engineered radiomic features^8,9,37^. However, to the best of our knowledge, few CADx studies have explored mpMRI analysis using deep learning. Dalmis et al. reported an approach of training a 3D CNN from scratch using three MRI protocols and patient information which yielded an AUC of 0.831 [0.791–0.867]^10^, while our study explored transfer learning and achieved better performance. Truhn et al. fine-tuned a pretrained residual neural network and achieved an AUC of 0.88^11^, whereas our study exploited feature extraction which is less computationally expensive and more suitable for small medical datasets. Note that the CNN input that yielded the best performance in Truhn et al. only contained information from DCE. Nonetheless, their approach was similar to the image fusion method in our study, which is not the optimal mpMRI scheme according to our results. Although additional information would be needed to statistically compare these results from the literature and ours, our approach demonstrated comparable and, in some cases, higher performance than others. We believe that our study is the first comprehensive study that investigated three different deep transfer learning schemes of exploiting multiparametric MRI information for lesion classification. The findings demonstrated superiority of one method, which can potentially inform future research in this field.

Our method extracts CNN features from the five max-pooling layers at the end of each convolutional block, average-pools, normalizes, and then concatenates to form CNN feature vectors^17,18^. Even though a more common way of applying a pretrained CNN to medical images is to extract features from fully connected layers at the end of the entire network architecture, the method requires image preprocessing to transform the images to a fixed size. Our method allows for using images of various sizes that correspond to enclosed lesion sizes and takes full advantage of the low- to mid-level features learned by the network.

When performing inter-sequence image registration, we chose to use multi-modality rigid registration that consists of translation and rotation. Scaling, shearing, or deformable registration was not employed because it was not desirable for quantitative image analysis to alter the geometry of and the texture within the lesions. More in-depth registration optimizations can be explored in future studies. Image registration can be computationally expensive and time-consuming. Given that all classifier performances were equivalent with or without image registration, we suggest that image registration might not be a necessary step in this proposed method of distinguishing between benign and malignant breast lesions using mpMRI.

A limitation of this study was the selection of the equivalence margin. The margin in equivalence testing is ideally a predetermined clinically meaningful limit. However, due to complexities and impracticalities in applying the statistical principles of equivalence testing to diagnostic performance studies, there is currently no widely used standard procedure to establish this margin^31^. Nonetheless, we were able to demonstrate equivalence between all classifier pairs using a rather conservative margin of 5% for ∆AUC. Furthermore, the pretrained CNN network requires 2D input, which limited the inclusion of the high-dimensional information contained in breast MRI exams. We chose to capture part of the 4D information in DCE-MRI by using second post-contrast MIP images in this study, and future work will include investigating the optimal approach to include high-dimensional information in medical images in deep transfer learning frameworks. Moreover, while not necessarily a limitation, we reported cross-validation performance scores instead of using a single training/validation/test split. Although a single split would be preferred if the data were abundant, we chose fivefold cross-validation to use the available data more efficiently and obtain high statistical power. It is important to note that the nested cross-validation scheme resulted in a 64%/16%/20% split into independent training, validation, and test sets within one partition in the outer cross-validation loop, and thus overfitting due to data leakage did not occur. In addition, the dataset for this study was moderately sized and was from a single institution, and therefore the model optimized on our dataset might not be the best solution on a different dataset from a different institution or population. The variation in image acquisition parameters in our dataset might also have impacted the results, but we believe that it positively contributed to the generalizability of the method to images acquired under different protocols.

In conclusion, our study proposed a mpMRI approach that significantly outperformed the CADx benchmark that uses DCE alone in the task of distinguishing between benign and malignant breast lesions. Our methodology is computationally efficient and does not require intensive image preprocessing. Future work will expand the analysis to include other valuable MRI sequences, such as diffusion-weighted imaging. In addition, while this study focused on the computational aspect of improving the performance of a CAD system, we would like to perform reader studies in the future to assess our system’s clinical significance when used as a secondary or concurrent reader for radiologists. Furthermore, by increasing the size of our database, the performance could potentially improve with a fine-tuned CNN and a standard training/validation/test split of the data instead of fivefold cross-validation. Finally, we intend to perform validation on independent datasets from other institutions in order to investigate the robustness of the methodology relative to imaging manufacturers, facility protocols, and patient populations.
